# Towards a reference material for microplastics’ number concentration—case study of PET in water using Raman microspectroscopy

**DOI:** 10.1007/s00216-024-05251-7

**Published:** 2024-03-28

**Authors:** Oliver Jacob, Elżbieta Anna Stefaniak, John Seghers, Rita La Spina, Gabriella F. Schirinzi, Konstantinos Chatzipanagis, Andrea Held, Håkan Emteborg, Robert Koeber, Martin Elsner, Natalia P. Ivleva

**Affiliations:** 1 Institute of Water Chemistry, Chair of Analytical Chemistry and Water Chemistry, Technical University of Munich, Lichtenbergstr. 4, 85748 Garching, Germany; 2 Joint Research Centre (JRC), European Commission (EC), Geel, Belgium; 3 Joint Research Centre (JRC), European Commission (EC), Ispra, Italy

**Keywords:** Microplastics, Reference material, Homogeneity, Stability, Raman microspectroscopy, Particle number concentration

## Abstract

Increasing demand for size-resolved identification and quantification of microplastic particles in drinking water and environmental samples requires the adequate validation of methods and techniques that can be used for this purpose. In turn, the feasibility of such validation depends on the existence of suitable certified reference materials (CRM). A new candidate reference material (RM), consisting of polyethylene terephthalate (PET) particles and a water matrix, has been developed. Here, we examine its suitability with respect to a homogeneous and stable microplastic particle number concentration across its individual units. A measurement series employing tailor-made software for automated counting and analysis of particles (*TUM-ParticleTyper 2*) coupled with Raman microspectroscopy showed evidence of the candidate RM homogeneity with a relative standard deviation of 12% of PET particle counts involving particle sizes >30 µm. Both the total particle count and the respective sums within distinct size classes were comparable in all selected candidate RM units. We demonstrate the feasibility of production of a reference material that is sufficiently homogeneous and stable with respect to the particle number concentration.

**Graphical Abstract:**

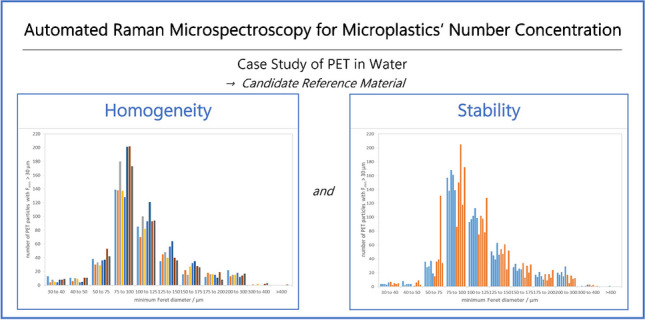

**Supplementary Information:**

The online version contains supplementary material available at 10.1007/s00216-024-05251-7.

## Introduction

Microplastics (MPs) have become one of the biggest analytical challenges in the last 20 years, not only due to a lack of a precise definition but also because of their constant alterations under environmental influence. The problem of defining what microplastics really are still involves many unanswered questions, despite around 20 years of scientific effort [1, 2].

The first attempt to address the problem of small plastic particles polluting the marine environment was DIRECTIVE 2008/56/EC (Marine Strategy Framework Directive, MSFD) [3], which laid the ground for comprehensive, large-scale actions in emerging areas, such as ‘properties and quantities of marine litter’ (Annex 1, Descriptor 10). The criteria “for good environmental status relevant to the descriptors of Annex I to DIRECTIVE 2008/56/EC” are outlined in the following *Commission Decision of 1.09.2010 (2010/477/EU)* [4]. Descriptor 10 (Annex Part B of the *Decision*) related to marine litter indicates the presence of microparticles and a need to characterise “trends in the amount, distribution and, where possible, composition of microparticles (in particular microplastics)”. This was tackled by the MSFD Technical Subgroup on Marine Litter (EC JRC); their *Guidance on Monitoring of Marine Litter in European Seas* [5], released in 2013, contains protocols and instructions on how to assess data on marine litter, including microlitter or ‘microplastics’, respectively. It was also emphasised that “an important part of establishing standard methods and protocols within MSFD will first be to define the appropriate size range”.

A comprehensive definition of MPs has been discussed since then, and it is still to be resolved. Although it is widely accepted that the upper limit should be set at 5 mm, the lower size limit applied in practice differs between various case studies (usually determined by a net or sieve mesh, filter pore size or the capacity of a technique in general). This lack of definition has been reflected—regarding the upper limit—in the *Commission Decision (EU) 2017/848 of* *17 May 2017*, “laying down criteria and methodological standards on good environmental status of marine waters (…)”, where the microlitter is described as *particles < 5 mm, classified in the categories ‘artificial polymer materials’ and ‘other’* [6].

Different stakeholders (standardisation bodies, policymakers, scientists, etc.) define microplastics according to their goals. ISO/TC 63/SC 14 (Plastics/Environmental aspects) released a Technical Report *ISO/TR 21960:2020 Plastics — Environmental aspects — State* *of knowledge and methodologies* [7]. They defined microplastics as “any solid plastic particle insoluble in water with any dimension between 1 and 1000 µm (= 1 mm)”, with two additional definitions of ‘large microplastic’ (1−5 mm) and ‘macroplastic’ (above 5 mm). Another definition has been created for the restriction on intentionally added microplastics, remitted by the European Chemicals Agency (ECHA) [8]. They were defined as “material consisting of solid polymer-containing particles, to which additives or other substances may have been added, and where ≥ 1% w/w of particles have (i) all dimensions 1 µm ≤ *x* ≤ 5 mm, or (ii), for fibres, a length of 3 µm ≤ *x* ≤ 15 mm and length to diameter ratio of > 3.” So far, there is no agreement among stakeholders about the final definition of microplastics.

Microplastics raised considerable public concern regarding their suspected effects on human health (together with potentially released endocrine-disrupting compounds and pharmaceuticals) through drinking water. They will, therefore, be put on a so-called ‘watch list’ according to the recast DRINKING WATER DIRECTIVE (DWD) [9], which entered into force in 2021, “addressing substances or compounds of concern to the public or the scientific community on health ground” (Article 13, paragraph 8). Now, there is ongoing work within the EC to define a methodology which would be the most suitable to determine the content of microplastics in tap water.

Along with a need for methodologies, there is a need for (matrix) reference materials (RMs, as defined in ISO 17034 [10] and ISO Guide 30:2015 [11]) with microplastics. RMs, inevitable for quality assurance and quality control in good laboratory practice, are used to verify the reliability of measurement systems. According to the definition, RM must be “sufficiently homogeneous and stable with respect to one or more specified properties, which has been established to be fit for its intended use in a measurement process” [11]. Certified reference materials (CRM) are complemented by a document stating the certified values associated with uncertainty and traceability.

It is quite challenging to validate analytical methods in some emerging areas, where CRM would provide the easiest and the fastest solution. In microplastic analysis, many attempts have already been made to produce reference materials, though not by accredited RM producers [12, 13]. In most cases, RMs for microplastics are homemade, as the one presented by von der Esch et al. (2020) [14]; thus, they are closer to quality control materials (QCM, ISO Guide 80 [15]). However, it must be emphasised that the term ‘reference material’ or ‘quality control material’ is sometimes used in publications to describe a material which properties are not assessed according to ISO 17034 and ISO Guide 80. All these issues highlight the effort around standardising analytical methods for MPs. The general need to determine microplastics, both qualitatively and quantitatively, has resulted in numerous publications with many different approaches to tackling validation in the analysis of MPs [16–22].

The challenges associated with producing microplastic RM are not only due to the lack of a clear definition and standardised analytical methods, but also to an ambiguous view on the impact on human health and the environment. It is rather challenging to choose a measurand if it is unclear which property of MPs should be monitored (polymer type, number concentration, mass concentration, particle size and shape, size distribution etc.). Therefore, the only reasonable solution is to apply an iterative approach, like developing simple RMs (i.e. pure standards) or simple matrix RMs (i.e. one analyte mixed with a simple matrix), which could serve in an inter-comparison or for internal quality control. These materials can then be used for the development of standard operating procedures (SOPs) and for the quality control/quality assurance of methods currently applied. With the more advanced and reliable measurements, the RM producers could prepare more complex RMs requiring more analytical skills. The last step would be a development of CRMs with assigned certified values. This whole complexity is sometimes called a ‘chicken or the egg’ problem since reference materials and standardised analytical methods cannot exist without each other. This problem has been thoroughly discussed by Emteborg et al. [23]. The RM producers need laboratories that can prove their ability to perform their analytical work according to ISO 17025 [24] and provide reliable results. The first approaches to test potential reference materials with MPs via interlaboratory study have already been reported in the literature [12, 13]. The results reported by JRC-BAM (Bundesanstalt für Materialforschung und -prüfung) [25] and QUASIMEME-Wepal [26], as well as the EUROqCHARM project (www.EUROqCHARM.eu), uncovered a strong necessity for standardised documentation of applied analytical and sample preparation methods as well as RM development.

This paper shows a case study of microplastic particles in a simple matrix (such as water) which would pave the way to produce a reference material with MPs’ number concentration as the measurand. The concept of this RM type has been described elsewhere [27]. A similar material, previously used for JRC-BAM comparison, was also investigated with respect to a potential characterisation (and certification, if applicable) of the PET mass concentration through ^1^H-NMR spectroscopy and gravimetry [28]. However, the determination of the particle number concentration is far more complex, even if only a simple matrix is considered. This has been revealed in the reports on the JRC-BAM interlaboratory study [25]. Here, we present a candidate RM of the kind as described in [25, 27, 28], which, however, has a lower PET particle number concentration. To assess the homogeneity and stability of a potential reference material with microplastics, an automated Raman microspectroscopy-based method has been used for the first time. This method enables the automated detection, quantification and morphological characterisation of (plastic) fragments in optical microscopy images, followed by automated Raman-based identification of MPs and non-plastic fragments, utilising the recently developed open-source program *TUM-ParticleTyper 2* [29]. The goal of this publication is to outline the skills required for a laboratory participating in the assessment of this specific RM, where not only particle counting but also size determination and chemical identity recognition in one individual measurement are needed. We aimed to illustrate that defining a measurand of RM with microplastics is not trivial, especially when size-resolved microplastic particle number concentration has to be analysed. As the size measurement must be performed according to a specific definition of the particle size, we also systematically addressed this topic. Based on the presented case study, we have demonstrated the obstacles and opportunities that appeared during the production and analysis of a potential (candidate) RM with MPs.

## Materials and methods

The candidate RM described below is based on the test material used for the JRC-BAM ILC on PET in water [25, 27, 28]. Each unit consists of pristine PET particles embedded in a sodium chloride layer, a surfactant solution and a bottle of demineralized water (Type 2), filtered through a 0.2 µm particle filter (Millipack, MERCK). The final material (water with PET particles) is ready to use after transferring the PET particles from the salt carrier into the water with the help of a surfactant solution. The role of surfactant is to decrease the interfacial tension between the hydrophobic particles and water. The three components are packed separately, and the reconstitution protocol is provided to help with the preparation of the final material. The idea of providing the three components separately was implemented to avoid the immediate separation of PET particles from water, which would compromise the homogeneity of the material.

Production of the candidate RM units (material quality control and processing) was done by JRC-Geel. The team from the Technical University of Munich, TUM (Institute of Water Chemistry, Chair of Analytical Chemistry and Water Chemistry, School of Natural Sciences, Technical University of Munich) performed the analytical part.

### PET particles

PET (polyethylene terephthalate) material, in the form of a powder (colourless, maximum particle size 300 µm), was purchased in the market (GOODFELLOW, Cambridge, UK). Its chemical identity was verified in bulk and on the level of individual particles. Bulk PET powder was characterised by FT-Raman spectroscopy using a Bruker Vertex 70 spectrometer (Bruker, Belgium) equipped with a RAMII module for performing the measurements. The spectrum was taken using an integrated air-cooled diode-pumped Nd:YAG excitation laser operating at 1064 nm with a liquid N_2_-cooled ultrahigh sensitivity Ge detector. Spectral collection was performed across a scanning range of 250–3500 cm^−1^ with 4 cm^−1^ resolution and a laser power of 450 mW; 64 scans were averaged with the OPUS software (OPUS, v.7.5.18) being used for data acquisition. The instrument quality check using a performance qualification (PQ) test was conducted prior to the measurement. The recorded spectrum presented in the Supplementary Material (SM, Figure S1) is in agreement with previous Raman reports on PET [30].

The purity of the PET particles was also checked at the particle level by dispersion in ethanol and drop-by-drop transfer onto a Si wafer. The individual particles were analysed with a confocal Raman microscope inVia (Renishaw, UK). The sample was placed on a motorised stage, and a preliminary identification test was performed to set the laser parameters and optimise the Raman signal. Secondly, a high-quality image of a large analysis area was acquired. This image was then processed with the particle analysis software. This step enables the instrument to register the position of the objects under investigation by using the contrast between the particles and the background. The objects were classified according to size and then automatically analysed with Raman to obtain the polymer spectrum. Data were collected using a 10× objective, 785 nm laser, 5% laser power (approx. 10 mW at the sample), 3 accumulations of 10 s. PET reference spectra were taken from the Renishaw spectral database and collected from the Polymer Kit 1.0 PET sample [31]. The Raman spectra of individual PET particles and their mappings can be found in the SM (Figure S.2).

### Processing

The stock suspension was prepared by mixing the PET powder and 0.05% Triton X 100 (p.a. grade, Merck) solution, stirred for 30 min and filtered through a 50 µm sonicated sieve (stainless steel, VWR, Belgium) to remove small particles. The particles collected on the sieve were transferred to a clean beaker and rinsed with ethanol. Dried PET particles were suspended in a mixture of 10% NaCl (p.a. grade, Merck) solution and 0.05% Triton X-100 solution, both filtered previously through a 0.22 µm pore size filter (Millipack, Merck). The particle number concentration and the size distribution were measured by a particle counter (PAMAS SVSS, Partikelmess- und Analysesysteme GmbH, Germany). The system was calibrated and maintained by the producer in the size range of 1–400 µm.

The aim was to achieve the number concentration of approximately 400 particles larger than 50 µm (sieve mesh) diameter. The production took place in a clean cell; all glassware and tools were cleaned in a dishwasher and rinsed with deionized water (Type 2, filtered through a 0.2 µm filter). The suspension with PET particles was stirred with an overhead stirrer, which kept the suspension in motion but without foaming. The vials, filled with PET suspension, were placed in a freeze-dryer at −40 °C. After drying, the vials contained a solid NaCl matrix with embedded PET particles and traces of Triton X-100. Transparent glass water bottles measuring 1 L were filled with deionized water (Type 2, filtered as mentioned above). The rinsing solution (0.1% v/v Triton X-100 in water) was also cleaned from particulates by filtering and then dispensed into 100 mL amber vials. The bottles with water were closed with polypropylene caps separated from the bottleneck by a metal disc. For more details on the production process, the reader is referred to Seghers *et al.* (2021) [27].

### Reconstitution

Before analysis, the water sample has to be reconstituted by transferring the particles from the NaCl carrier (a) into water (950 mL) (b) using the provided surfactant (c). Every candidate RM unit consists of these three components (a), (b) and (c). The reconstitution protocol is attached to each unit and must be followed every time the candidate RM is to be used. The vial with the particles embedded in NaCl (a) is filled with 5 mL of the surfactant (c), shaken gently, and the content is poured into the water (b). This step is repeated eight times; one additional 5 mL portion has to be used to rinse the vial cap. In total, 50 mL of the surfactant solution (c) must be used to obtain 1 L of the water sample with PET particles. Plastic parts and tools must be avoided as much as possible (replaced by glass or metal) and the reconstituted sample has to be analysed immediately.

### Filtration

After reconstitution, the water samples were filtered under vacuum (∆*p* = 250 mbar) on Au-coated track-etched polycarbonate filters (pore size 0.8 µm, Analytische Produktions-, Steuerungs- und Controllgeräte GmbH, Germany), using a Sartorius filtration apparatus 16306 (25 mm glass vacuum filter holder, glass frit filter support, Sartorius, Germany). All steps were carried out in a laminar flow box (ENVAIR eco, ISO 3 standard, ENVAIR GmbH, Germany). After filtration, the filtration apparatus and the 1 L water bottle were rinsed with MilliQ water (3× 50 mL), which had been additionally filtered over a cellulose acetate filter (pore size 0.45 µm). An additional 50 mL portion was used to rinse the filtration apparatus only. Ten laboratory blank samples were collected during the sample processing, in each case immediately before filtration of the sample. Here, 200 mL of water (corresponding to the 4 × 50 mL rinsing portions described above) was used only (MilliQ, additional filtration over cellulose acetate filter, pore size 0.45 µm, from the same source as the one used for rinsing the samples and the filtration system). After collecting the blank sample, the filtration apparatus remained unchanged.

### Raman measurements

Chemical identification was performed by Raman microspectroscopy (WITec apyron, WITec GmbH, Germany) using a 532 nm laser with 4.5 mW power. The settings of Raman signal collection were as follows: maximum spectral acquisition time 40 × 0.5 s, minimum required signal-to-noise ratio (SNR) of 5, spectral autofocus ranged from −10 to +360 µm. For the measurement series “Evaluation of small particles’ content”, an objective with 100× magnification, NA = 0.9 (working distance, WD = 1.0 mm), was used (together with the alternative spectral autofocus range from −10 to +15 µm). For all other measurement series, an objective with 20× magnification and NA = 0.4 (WD = 2.2 mm) was used. The spectral range used for observation was 150–4000 cm^−1^, and for comparison with the reference spectrum (database matching), it was 590–1770 cm^−1^ and 2800–3200 cm^−1^ (Raman shift). The Raman system was calibrated with external (Si wafer) and internal (diode lamp) standards. The Raman signal intensity was checked regularly with the Si band at 520 cm^−1^.

PET particles were analysed and counted by static image analysis followed by automated Raman measurements using the homemade open-source software *TUM-ParticleTyper 2* [29]. The particles down to 10 µm were studied on the entire filter using “random sampling”. The mode “random window subsampling” was used to measure particles in the size range 1–50 µm. In this mode, objects are detected and chemically identified within randomly distributed small areas only (observed filter area proportion 0.5 to 1%). After image preprocessing, the functions cv.adaptiveThreshold(), cv.findContours() and cv.minAreaRect(), all from the Python project OpenCV, were used to outline the particle perimeter and particle area. From the latter, the smaller dimension of the minimum bounding rectangle (MBR) was considered as an approximation of the minimum Feret diameter.

## Results and discussion

The number concentration of PET particles in a certain size range is the measurand, which value had to be determined. According to VIM 200:2012, measurand is ‘a quantity intended to be measured’ [32]. Further in [32], it is noted that ‘the specification of a measurand requires knowledge of the kind of quantity, description of the state of the phenomenon, body, or substance carrying the quantity, including any relevant component, and the chemical entities involved’.

In the case of our candidate RM, it was necessary to define a way to express a particle size class. For spherical particles, the definition of a particle size would be straightforward. However, particle sizing will always be an operationally defined measurand, as the size definition depends on the physical principle applied for its measurement [33]. When irregularly shaped objects are to be considered, various approaches are available for indicating the size. Measuring a particle size through microscopy will apply a projection diameter (two-dimensional image of a particle), which implicates a limited choice for size definition: Feret diameter, the dimensions of the minimum bounding rectangle (MBR), the equivalent projected area diameter (circular-equivalent diameter, CED), or the Martin diameter. The first three have found a wide application in microscopic research.

The difficulty of defining the measurand for irregular particles stems not only from a problem of selecting a size definition, but also from a practical approach to its measurement. Although many microscopes combined with vibrational spectroscopy are equipped with software for automatic size measurement and counting particles, there is often no option to obtain the Feret diameter or the CED. The software sometimes labels the particle dimension as ‘width’ or ‘length’. This can probably be associated with the dimensions of an object’s minimum bounding rectangle (MBR). Besides easily available freeware (i.e. ImageJ), which can be used for this purpose, many research groups also create and apply their own software to process a digital projection of a particle and to extract its parameters [34]. The commercial software *WITec ParticleScout* returns, among others, values for maximum and minimum Feret diameter, the dimensions of the MBR and CED. Based on the particle projections of approx. 800 PET particles from the source described above [25], Table 1 shows the relative difference between these size measures and the minimum Feret diameter (*F*_min_), considered the most appropriate in the following. If the size distribution based on *F*_min_ is changed to an alternative basis for particle sizing, it will be shifted according to the percentage values given.

**Table 1:** Relative differences of selected size measures referred to the minimum Feret diameter. Here, all deviations are given as 1 SD (standard deviation).

Size class / µm	Rel. diff. of MBR* length to *F*_max_^†^		Relative difference to *F*_min_^‡^					
			Width of MBR		*F*_max_		*CED*^§^	
30 to 40	−2.9%	± 4.3%	1.1%	± 2.7%	90.0%	± 56%	30.1%	± 23%
40 to 50	−0.8%	± 3.3%	0.1%	± 0.5%	147.8%	± 100%	38.4%	± 28%
50 to 75	−3.3%	± 4.7%	1.3%	± 3.5%	75.1%	± 42%	20.1%	± 14%
75 to 100	−4.8%	± 5.6%	1.6%	± 3.6%	64.0%	± 41%	13.2%	± 14%
100 to 125	−5.5%	± 5.7%	1.9%	± 4.1%	54.4%	± 31%	9.5%	± 11%
125 to 150	−5.3%	± 5.2%	1.0%	± 1.6%	55.4%	± 31%	7.3%	± 13%
150 to 175	−6.9%	± 7.0%	4.3%	± 4.6%	52.4%	± 22%	4.4%	± 8.4%
175 to 200	−8.5%	± 7.7%	3.9%	± 5.4%	80.4%	± 125%	6.6%	± 14%
Complete range	−4.5%	± 5.5%	1.6%	± 3.5%	67.0%	± 45%	14.7%	± 15%
□	−29.3%		0		41.4%		12.8%	
∆	0		0		15.5%		−14.3%	

*Minimum bounding rectangle

†Maximum Feret diameter

‡Minimum Feret diameter

§Circular-equivalent diameter

Simple geometric objects (square, “□”, and triangle, “∆”) can show significant differences between these measurements, as is the case for the longer side of the MBR and the maximum Feret diameter. However, within “real” samples, this difference gets significantly smaller. Additionally, the smaller dimension of the MBR is generally suitable as an approximation for *F*_min_, whereas the CED gives a considerably higher deviation in this respect.

**Fig. 1:**
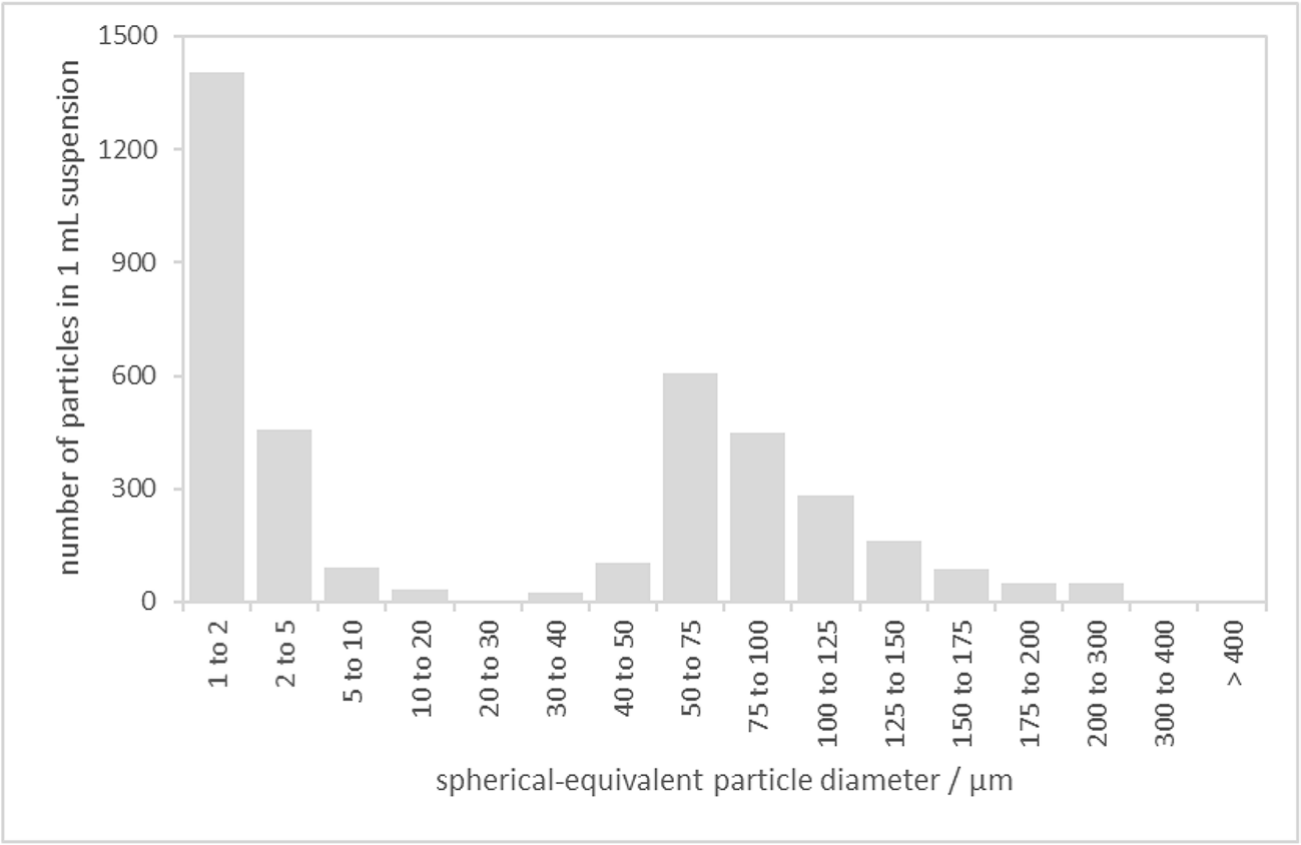
Particle size distribution in the PET stock suspension, as measured by a particle counter

Figure 1 shows a number-based particle size distribution of the stock suspension, determined by a particle counter. This technique is based on the light obscuration phenomenon, and the particles are sized according to their hydrodynamic diameter. Within the size range of interest (30 to 1000 µm), the mode is located at the size class 50–75 µm. Within smaller size ranges (1–10 µm), the expected increase in particle counts can be observed. However, particles within this size range contribute only marginally to the overall particle mass and can be excluded from the analysis using particle counting methods.

Application of microscopic techniques to determine the number of particles means that the basic method applied here is counting, expressed as a ‘number of entities’. The result will always be based on the identity of these entities (size range, shape, aspect ratio, chemical identity etc.) and will be affected by the uncertainty of the methods for implementation of these underlying input quantities. In the case of plastic microparticles, this must involve not only sizing and counting but also recognition of a polymer type. Therefore, techniques combining microscopy with a chemical detection identity system seem the best choice. So far, Raman and Fourier transform (FT) infrared (IR) microspectrocopy are often used for that purpose [35, 36]. However, with respect to (C)RM development, these analytical methods have to be first standardized and validated.

Another issue affecting the size measurement is the lateral resolution, which mainly depends on the magnification and numerical aperture (NA) of the applied objective and additional limiting factors like (possibly lower) pixel resolution achievable through software. The lower the particle size, the higher the (physical) lateral resolution should be. To measure particle sizes down to 1 µm, high magnification was needed (objective 100×, NA 0.9), which leads to the resolution of 8.86 pixels/µm, or a pixel size equal to 0.112 µm, thus passing the minimum requirements set by *TUM-ParticleTyper 2* for the area of an object (>13 pixels). On the other side, an acceptable precision and a limit of quantification at the lower double-digit range of particle counts would require very time-consuming and tedious measurements. For the analysis of particles ≥10 µm, an objective with 20× magnification was used, which leads to a resolution of 0.81915 px/µm (1 pixel = 1.2207 µm), indicating a particle sizing precision of approximately 1 µm. The achievable precision of the size determination affects the precision of the counting result differently depending on the particle density at the lower limit of the size range to be considered.

This leads us directly to the question of the appropriate size range itself. The idea was to produce a material with larger PET particles, i.e. from 50 µm upwards. As already shown above (Fig. 1), it was possible to diminish the number of smaller PET particles in the suspension used for production, but it was not possible to eliminate all small particles. The smaller the size, the more particles can be found in the suspension and, therefore, in the final material. Based on the results for particle size distribution shown above, it was decided to measure the number of all PET particles larger than 30 µm. By variation of this cut-off diameter, we could then easily determine the effect on the PET particle number concentration to be determined, since the particle size, expressed as minimum Feret diameter *F*_min_, is known at the level of individual particles.

To become a reference material, the candidate RM had to be assessed according to the requirements of ISO 17034. Both homogeneity and stability (storage and transport) were evaluated by analysis of PET particle number concentration in the allocated units.

### Determination of PET particle number concentration and particle size distribution for homogeneity assessment

Homogeneity assessment aims to prove that all the units in the whole batch of a reference material contain equal amounts of the analyte within the range of uncertainty. The number of units to be analysed is based on the total number of units in the batch, and (as a guideline) it is usually the cubic root of the total number of units in the batch. According to ISO Guide 35:2017, “a subset of units, typically 10 to 30, is chosen from the batch using a suitable sampling scheme” [37]. These units must be selected in a randomized way, covering the whole batch of produced units.

Raman microspectroscopy, in combination with the open-source software *TUM-ParticleTyper 2* [29], was used for the analysis of ten units allocated to homogeneity assessment. This method of chemical identification and particle counting has been described elsewhere [14, 29, 34, 35]. Additionally, the repeatability of the counting has been tested using one reconstituted and filtered water sample of this material. Six replicates of particle counting (including the size measurement and chemical identification) on the same filter indicated a relative standard deviation of PET particle numbers of 0.63%. However, this very high repeatability does not comprise the sample preparation (filtering), which in the case of microplastics, is extremely challenging and contributes largely to the method uncertainty.

The mean value of PET particle numbers in the units assigned to homogeneity assessment is 411, with an SD equal to 50 and an RSD of 12.1% (*n* = 9, as one from 10 analysed units was rejected due to justified technical reasons). The individual results are shown in the SM, Table S.1. As mentioned above, comparing the particle counts when changing the lower size limit by 1 µm helps estimate the possible shift of the final result, which is caused solely by the chosen image resolution (between 0 and 2 particles per sample). In principle, a systematic error in particle size measurement must also be assumed, although its determination is intricate. A realistic impact may be represented by the particle counts within the size class 30–40 µm or 20–30 µm, respectively. This will be discussed further in “Evaluation of small particles’ content”.

The results of blank measurements at the same size range are in Table S.2 (SM). No contamination from PET was detected in any case; hence, the appropriate preconditions for the analysis of PET particles are met. Other polymers were present only in negligible amounts. The principle of one blank per analysed sample was maintained for all measurement series presented in this study.

**Fig. 2:**
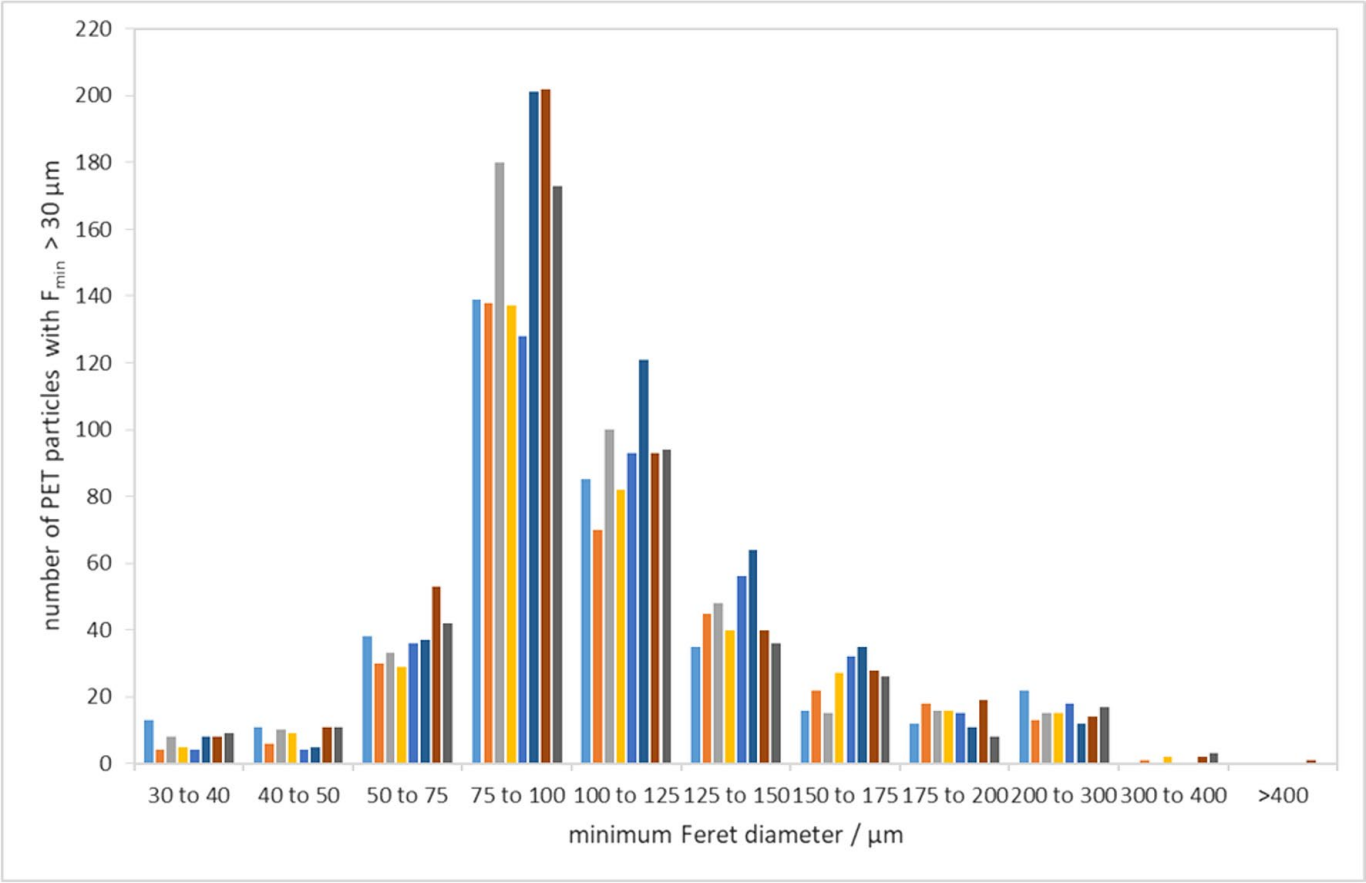
PET particle size distribution in the candidate RM units assigned to the homogeneity study (only eight units were used in this graph; each colour represents one unit)

The size distribution of PET particles in analysed units determined by Raman-based method (Fig. 2) is similar to the size distribution in the PET stock suspension (Fig. 1) but shows the maximum in the size range 75–100 µm (instead of 50–75 µm). Furthermore, the number of particles in the selected size bins varies largely; narrowing down the size range, e.g. from 50 µm upwards, would not decrease the RSD of the PET particle number. On the contrary, it reaches 13.2% (*n* = 8, as particle size distribution was not available for all units). For particles above 75 µm, it is 13.4% and above 100 µm, it appeared to be 12.7%.

### Analysis of units allocated to stability assessment

A stability study can answer the question of whether the material remains stable during storage and transport. This could either be reached through isochronous studies (the units assigned to the study are kept at both reference and test temperatures for a certain period and then analysed altogether under repeatability conditions) or through a stability assessment of a similar material, which, however, has rather limited validity. Another way could be using literature data and common knowledge.

**Fig. 3:**
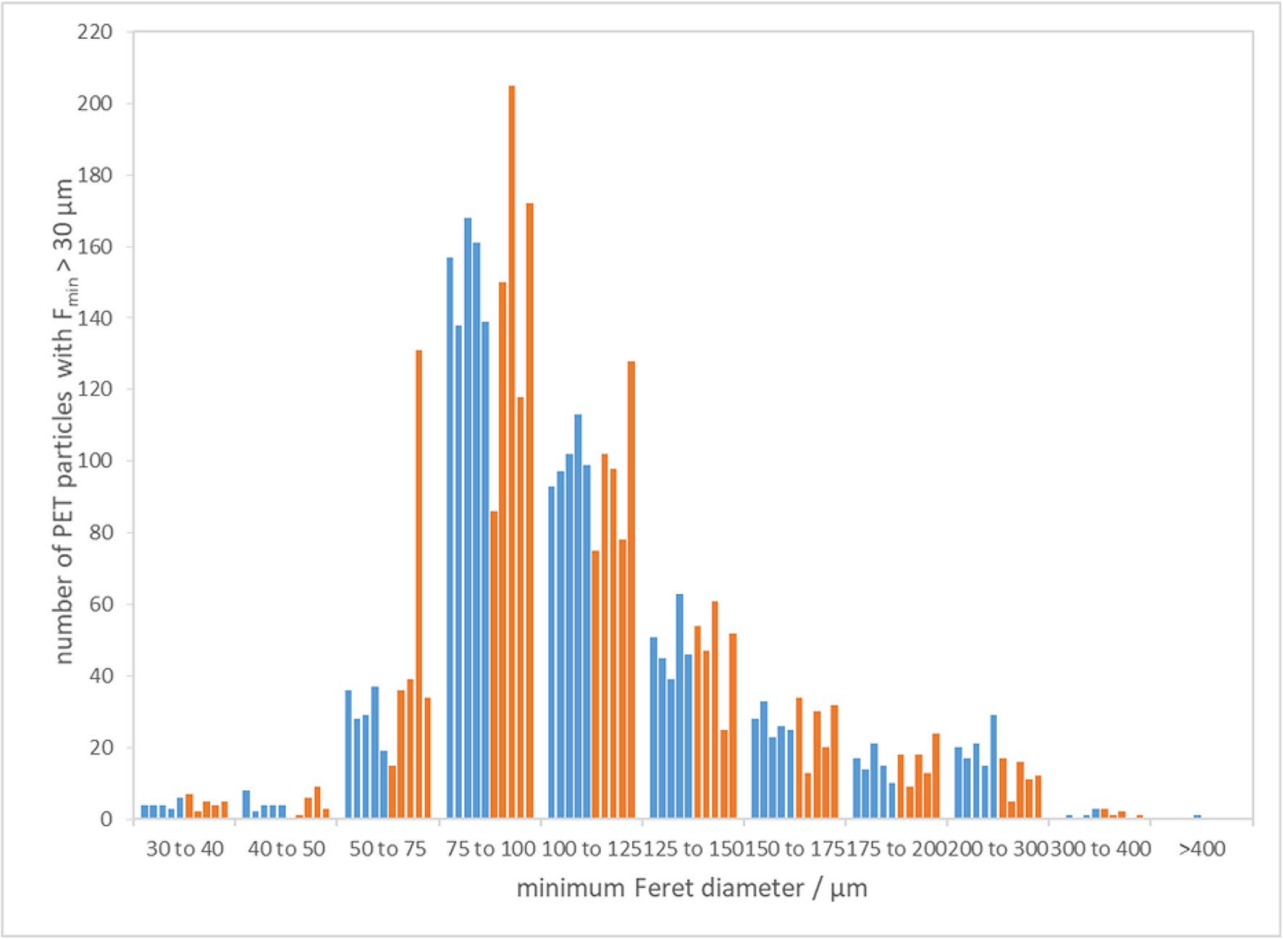
PET particle size distribution of the candidate RM units kept at reference temperature (blue) and exposed to transport conditions (orange).

Transport stability is assessed for (C)RMs, which might decompose due to extreme conditions caused by transport. Ten units were allocated for this study: five were subjected to transportation in the trunk of a car for 4 weeks, whereas the other five were kept at 4 °C in a fridge during this period. During transport, the highest temperature recorded by a data logger (placed together with the transported vials) did not exceed 35 °C. Additionally, the vials subjected to transport conditions went through a vertical impact test by dropping (1.5 m height). Afterwards, all units were analysed the same way as the ones assigned to the homogeneity assessment. The number-based size distribution of PET particles is presented in Fig. 3; more detailed results are shown in Table S.3 (SM). The results of blank analyses are presented in Table S.4 (SM). Again, no contamination from PET was detected in any case, and other polymers were present only in negligible amounts.

The average PET particle number in the units exposed to transport conditions (*n* = 5) and stored at reference temperature (*n* = 5) was in both cases, 405. However, the values for the transported units are more scattered, so the RSD values for both groups, i.e. units exposed to transport and kept at 4 °C, are 17.3% and 6.1%, respectively. The size classes of PET particles are equally populated, as the particle size distribution does not show any significant deviation from the one in the stock suspension (see Fig. 1 for comparison).

**Fig. 4:**
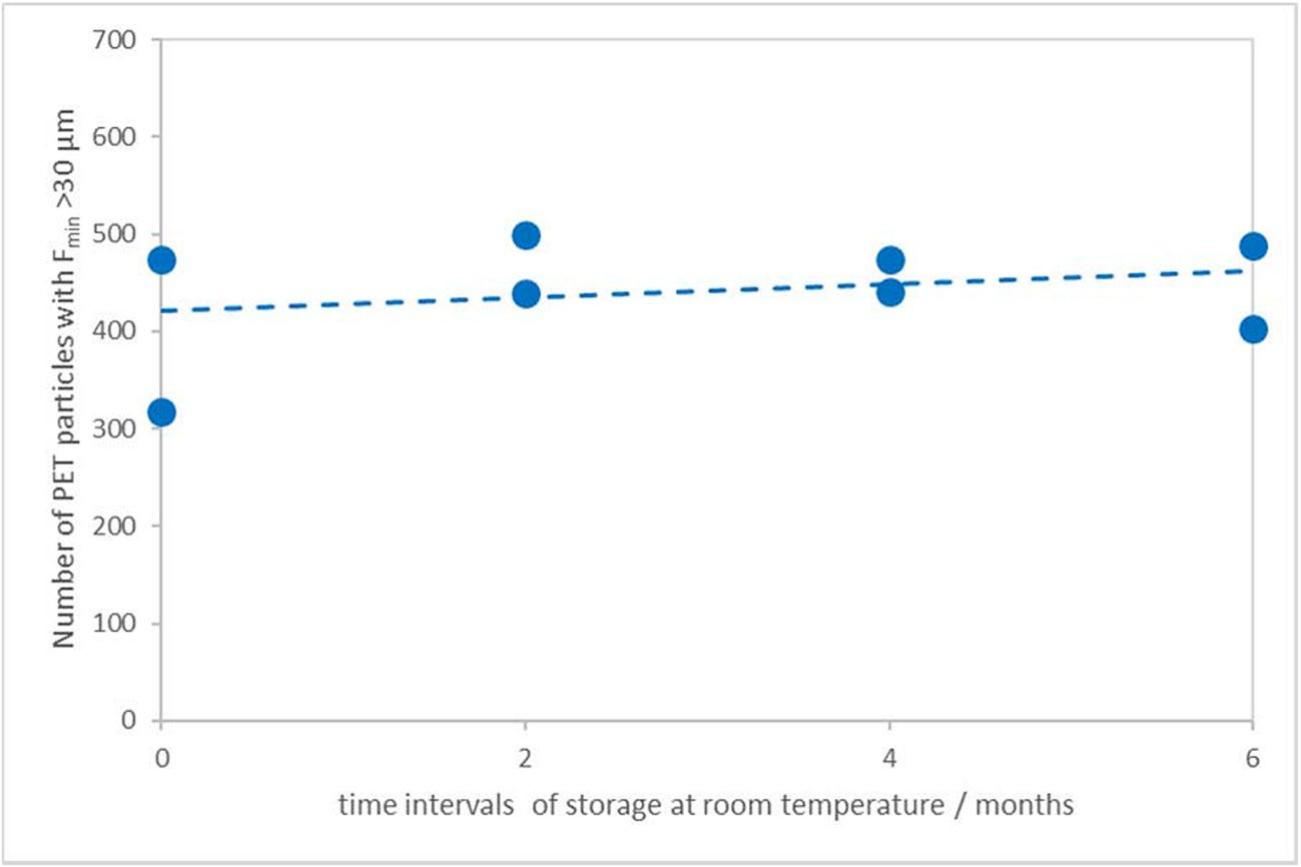
Average PET particle number concentration of the candidate RM units assigned to storage stability assessment

Storage stability was assessed via an isochronous study, with the same number of units stored at room temperature (RT) for different time intervals (therefrom zero to 6 months). Simultaneously, other units were kept at reference temperature (4 °C). The averages of the PET particle number concentration during the varying times of storage at RT remained stable; no significant changes in the particle numbers could be observed (Fig. 4). The dashed line (regression line) represents the trend (drift) of the values of PET particles’ number concentration. The slope was tested towards a statistically significant trend at a 95% confidence level and it appeared non-significant. Thus, the material can be stored at RT and transported without cooling elements. The detailed results are presented in Table S.5 (SM); the accompanying blanks show no signs of contamination (Table S.6, SM).

**Fig. 5:**
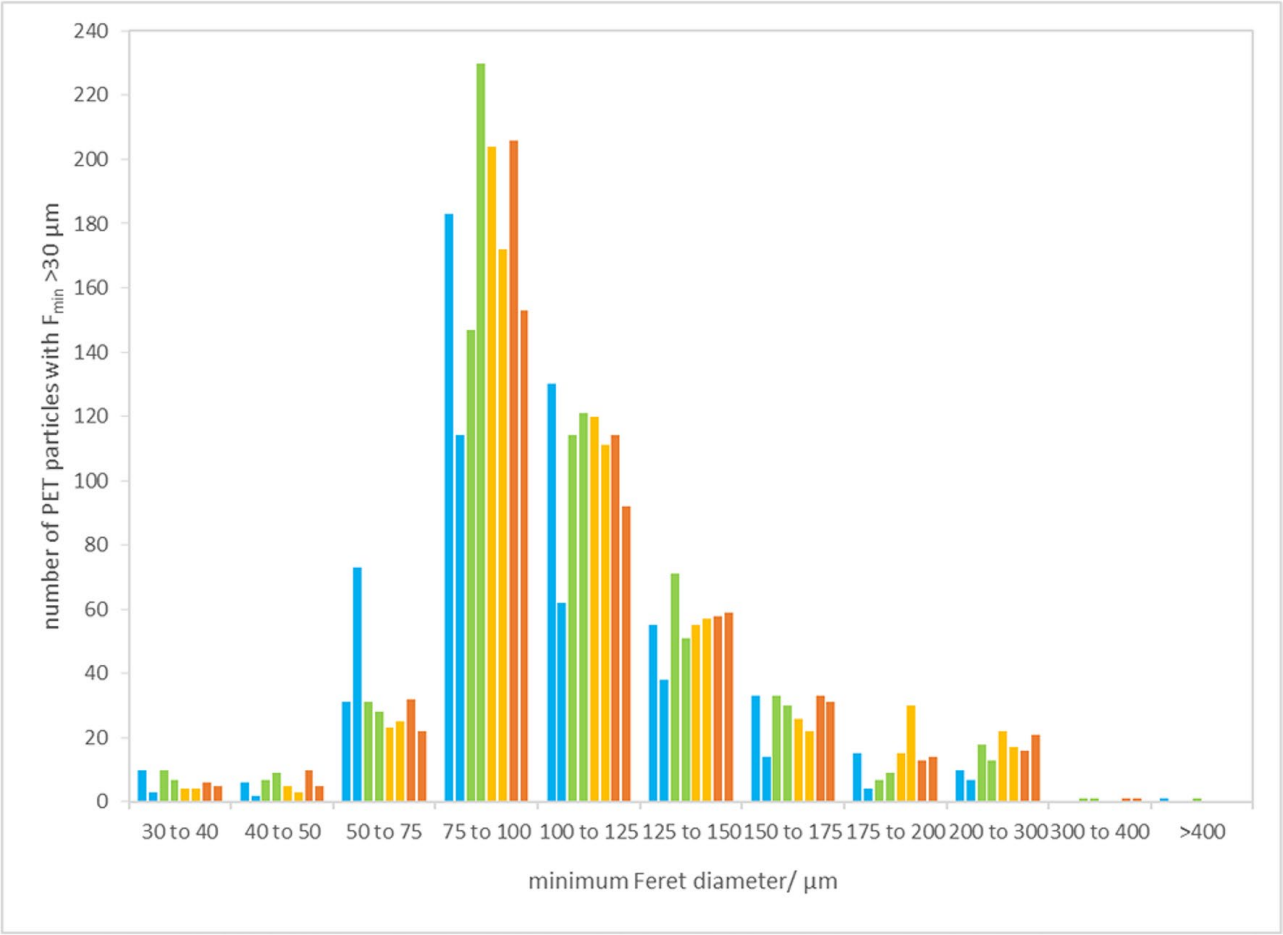
PET particle size distribution in the units assigned to storage stability assessment. Different colours are related to different time intervals of room temperature storage

The size distribution of PET particles (Fig. 5) of the units assigned to storage stability assessment is consistent with the other units used for homogeneity and transport stability.

### Evaluation of small particles’ content

All particle counts reported above were linked to the size range set from 30 to 1000 µm defined at the beginning. The goal here was to evaluate the content of particles below the cut-off limit (30 µm). Six randomly selected units were also analysed in a randomised order, to characterize particles equal to and below 50 µm down to 1 µm. For this purpose, every filter had to be analysed twice: first, with the Raman microscope set up for the size range 10–50 µm, where all particles were measured (20× magnification objective). Secondly, the filter was analysed for particles of the size range 1–10 µm (100× magnification objective), with larger particles being excluded by the software. As particle counts usually increase exponentially towards smaller sizes, it is impossible to measure all particles anymore. For that purpose, the Random Window Sampling algorithm was applied. The procedure is presented in detail elsewhere [29]. With this approach, a small part of the whole filter surface was analysed by image recognition and subsequent Raman measurements by analysing randomly chosen windows successively. The software extrapolates the total number of particles in the 1–10 µm size range from all window results. The number of concentration values and blanks’ composition can be found in the SM (Tables S.7 and S.8). As the Raman microscope’s capacity is limited to an amount of about 3000 particles within 20 h, the precision of any result obtained with this approach is bound to the measurement time one is willing to spend for the analysis. The extrapolation factor resulting from the proportion of the measured filter area also defines the step size for counting, which could not undercut the lower three-digit range due to the chosen measurement time per sample. Due to this limited precision consistent with the choice of applicable measurement times (max. 24 h per sample), the particle counts within the size range 1–10 µm ranged between 500 and 1269, with a median of 807. The average value of PET particle counts within the size range 10–50 µm (*n* = 6) is 25 with a RSD of 32%. Due to its relation to this lower average value (when compared to > 400 PET particles found in the context of the studies presented above), the RSD basically has a rather limited comparability to the uncertainty of 12% resulting from the homogeneity study. The PET particle size distribution of the allocated units can be found in Figure S.3.

**Fig. 6:**
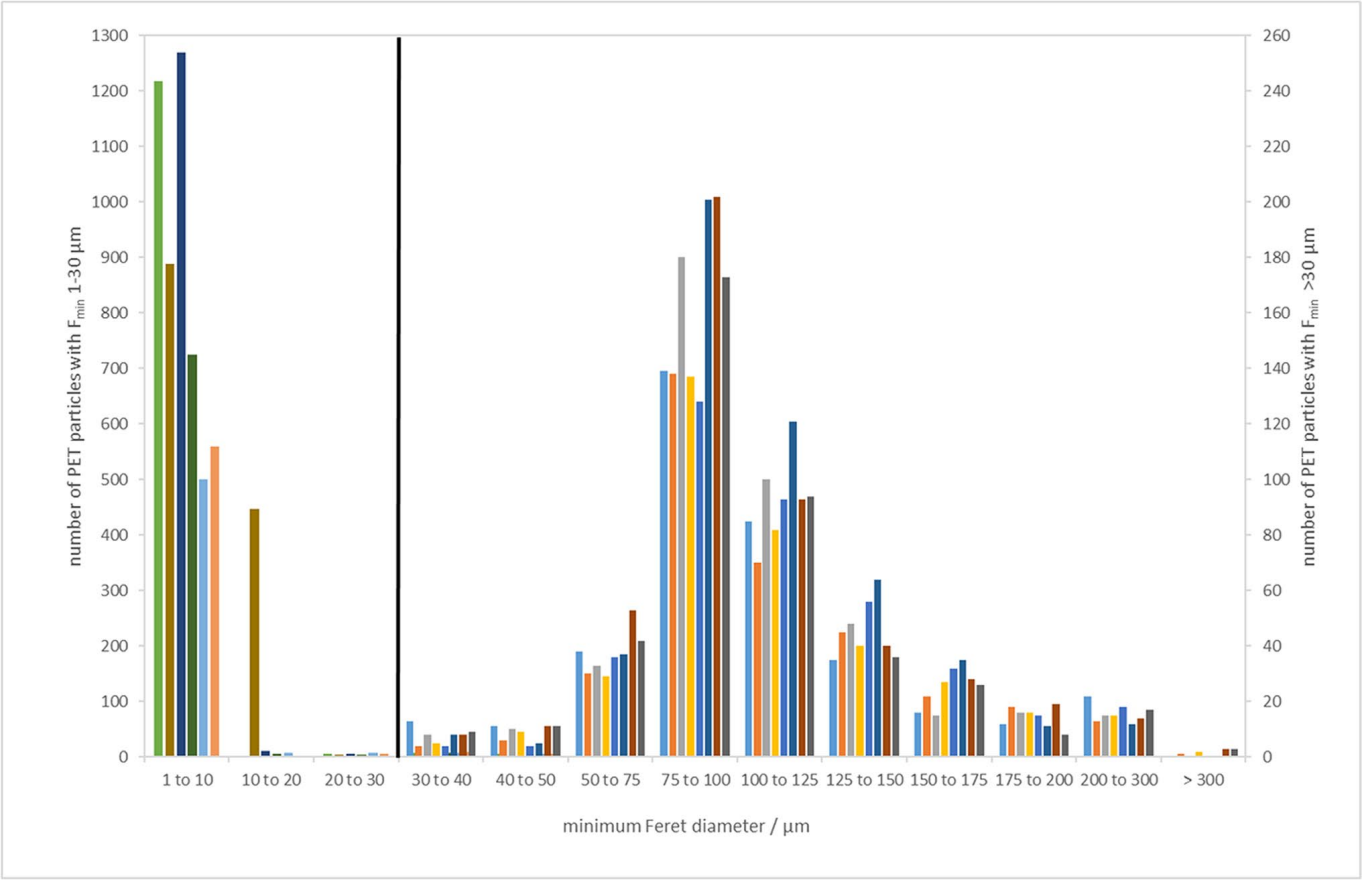
PET particle size distribution in the candidate RM units assigned to both evaluation of small particles’ content and homogeneity. The number of particles in the size range 1*–*10, 10*–*20 and 20*–*30 µm are referred to the left axis; the number of particles of the size 30*–*1000 µm - to the right axis

The PET particle size distribution in the size range of 1–1000 µm can be derived by combining the results from the evaluation of small particles’ content and homogeneity assessment. The particle counts for the size range 1–30 µm and those for the size range 30–1000 µm from the homogeneity study are presented side by side in Fig. 6.

It is worth noticing that the number of PET particles in the size range of 30–50 µm fits the pattern presented by all other units of the material, assigned to homogeneity, stability and evaluation of small particles’ content. The ‘zoom-in’ part of the size distribution, presenting only the size bins 30–40 µm and 40–50 µm, is shown in Figure S.4 (SM).

### Presence of non-PET particles

Despite all the measures taken to prevent cross-contamination with non-PET particles, each unit of the candidate RM material contains ‘foreign’ particles, which are made either of other polymers (PS, PE, PP, PTFE etc.) or of natural origin materials, i.e. cellulose, skin, CaCO_3_ etc. Techniques facilitating chemical identity recognition, particle counting and sizing consequently enable a resolution in this regard. ‘Pure’ counting and sizing techniques (optical microscopy, particle counters etc.) cannot confirm an unchanged composition between single units of the candidate RM regarding the involved bulk material.

Another aspect related to the presence of non-PET particles in the candidate RM is the mass control of PET. Considering the mass of microplastics being a potential measurand in a newly designed RM, it must be considered that non-PET particles might contribute to the total mass of the particles filtered out of the reconstituted water sample. We decided to estimate how much the ‘foreign’ particles would contribute to the total mass of PET particles per unit. Raman microspectroscopy (coupled with suitable software) enables not only measuring the size (Feret diameter) or area of individual particles but also an (manually driven and optically based) estimation of their thickness. Table S.9 (SM) shows examples of non-PET particles recognized in the candidate RM units allocated to homogeneity.

Based on the information from Table S.1 (SM) and using an average density value for each recognized polymer (adopted from [38]), we were able to approximate an average mass of non-PET particles per unit (*n* = 8), as presented in Table 2. The density of dust particles was set as 1.87 g/cm^3^, following [39]. As it can be concluded, the average mass of both not-PET polymer and dust particles per unit is barely 0.25% of the average mass of PET particles. Certainly, it might be possible to determine the contribution of other polymers (other than PET) to the total particle mass per unit. However, it would require an advanced technique such as pyrolysis-GC/MS, TED-GC/MS etc. On the other hand, due to a diverse signal intensity, the total mass of non-PET particles with respect to the mass of PET makes the analysis very difficult.

**Table 2:** The number and estimated mass of non-PET particles in the candidate RM units allocated to homogeneity study.

Unit	Number of PET particles with Feret_min_ diameter ≥31 µm	Number of non-PET polymer particles with Feret_min_ diameter ≥31 µm	Mass of non-PET polymer particles with Feret_min_ diameter ≥31 µm [µg]	Number of not identified (dust) particles with Feret_min_ diameter ≥31 µm	Mass of not identified (dust) particles with Feret_min_ diameter ≥31 µm [µg]	Total mass of non-PET particles with Feret_min_ diameter ≥31 µm [µg]
1	370	8	0.39	8	0.34	0.73
2	345	0		1	n/d*	
3	424	1	0.11	2	0.05	0.16
4	361	3	0.24	0		0.24
5	384	1	0.05	3	0.69	0.74
6	426	3	4.35	8	0.26	4.61
7	494	3	0.17	3	0.06	0.23
8	470	8	0.61	4	0.21	0.82
9	419	2	0.08	6	0.14	0.22

*no thickness determined

Non-PET particles of natural origin (Table S.1, SM) comprised a notable population in the candidate RM units, and their presence is inevitable. They mainly originate from sources like paper (cellulose), human skin, Earth’s crust or tap water droplets. Although the availability of both maximum and minimum Feret diameter and, even more relevant, the projection area of each particle, the absence of information on the thickness considerably complicates the estimation of their total mass. In view of their low density (tissue: 0.25–0.50 g/cm^3^, paper 0.5–0.8 g/cm^3^ [40]), their contribution to the total particle mass is negligible.

## Conclusions and prospects

Certified reference materials with microplastics applicable for any method of quantifying all particles with a specific chemical identity are not yet available. For a certification procedure of a candidate reference material, accepted validated methods are inevitable. However, for the validation of methods, suitable reference materials would be very useful. We have demonstrated that the candidate RM presented here fulfils the definition of a non-certified reference material, since the assessment of homogeneity and stability (both transport and storage) was satisfactory with respect to the number of PET particles and their size distribution. However, the possibility of developing a CRM based on this material, with a certified value of the microplastics’ number concentration, is still limited. At first, an appropriate lower size limit must be selected; this was not trivial considering the high population of small-sized particles typically present in the samples. The evaluation of particle size distribution in the stock suspension showed indeed a significantly lower number of particles in the size range of 20–40 µm, but they were not eliminated. Moreover, a contribution from the particles of the size range 1–10 µm to the total count was substantial. Taking into account all particles of the size above 30 µm, the average particle number in the units assigned to homogeneity was assessed with a relative standard deviation of 12.1%. As only low PET particle numbers in the size range between 20 and 40 µm were observed, the contribution of the particles of a size close to the cut-off diameter (e.g. 30–31 µm) is negligible. The uncertainty of size determination based on the image resolution itself (~ 1 µm) has, therefore, only a marginal impact on the counts (0–2 particles referred to the size range 30–400 µm). Transport stability assessment proved no significant deviation between the assigned units, neither for the total count nor for the size distribution of PET particles. Moreover, the candidate RM appeared stable at room temperature. We also indicated the capabilities required for a proper determination of microplastics in a water matrix, with respect to participation in RM assessment—in particular the simultaneous determination of size expressed in a certain way, automatic particle counting within the selected size range and recognition of the chemical identity of each particle. It has been shown that Raman microspectroscopy combined with appropriate software (for chemical identification, sizing and counting of particles) is suitable without restriction for this purpose. An additional ability to determine the particle thickness, along with its size and area, could enable an estimation of the contribution of non-PET particles to the total volume (or mass) of particles per unit. In conclusion, this candidate reference material could become another step further in the iterative approach towards certification of the microplastics’ number concentration in a water matrix. Along with the property value assignment, as required in the certification process, the traceability statement must be provided, which—in the case of this specific type of materials—will be an additional challenge. Therefore, a stepwise solution, i.e. sequential improvements of both candidate RMs and analytical methods, is the most suitable strategy.

### Supplementary Information

Below is the link to the electronic supplementary material.

Supplementary file1 (PDF 440 KB)
