# Supplementary material for: Towards a reference material for microplastics’ number concentration—case study of PET in water using Raman microspectroscopy
Source: Anal Bioanal Chem. 2024 Mar 28;416(12):3045–58. doi: 10.1007/s00216-024-05251-7 (PMC11045626; doi:10.1007/s00216-024-05251-7)
Supplement: Supplementary file 1 — Supplementary file1 (PDF 440 KB) [file 216_2024_5251_MOESM1_ESM.pdf]

SUPPLEMENTARY MATERIAL (SM)

Towards a reference material for microplastics' number concentration – case study of PET in water using Raman microspectroscopy

Oliver Jacob[1,†], Elżbieta Anna Stefaniak[2,†], John Seghers[2], Rita La Spina[2], Gabriella F. Schirinzi[3], Konstantinos Chatzipanagis[2], Andrea Held[2], Håkan Emteborg[2], Robert Koeber[2], Martin Elsner[1], Natalia P. Ivleva[1,*]

[†]shared first authorship

[1]Institute of Water Chemistry, Chair of Analytical Chemistry and Water Chemistry, Technical University of Munich, Lichtenbergstr. 4, 85748 Garching, Germany

[2]Joint Research Centre (JRC), European Commission, Geel, Belgium

[3]Joint Research Centre (JRC), European Commission, Ispra, Italy

*corresponding author: natalia.ivleva@tum.de

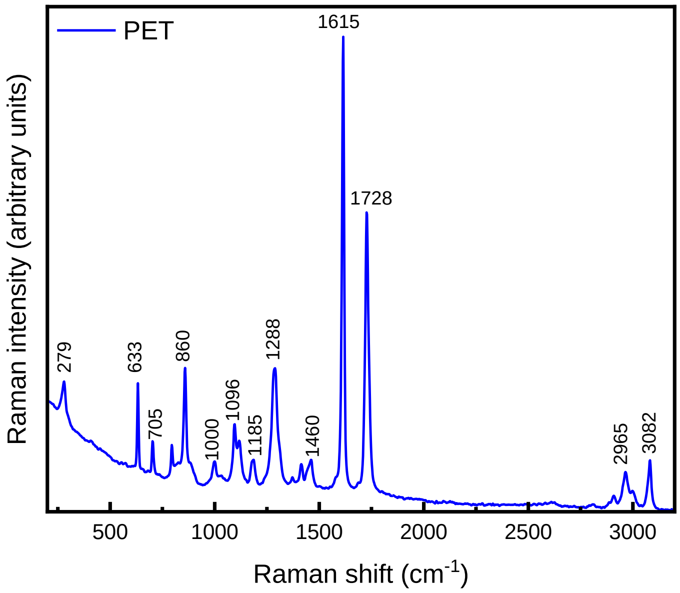

Figure S.1: FT-Raman spectrum of PET material used for RM.

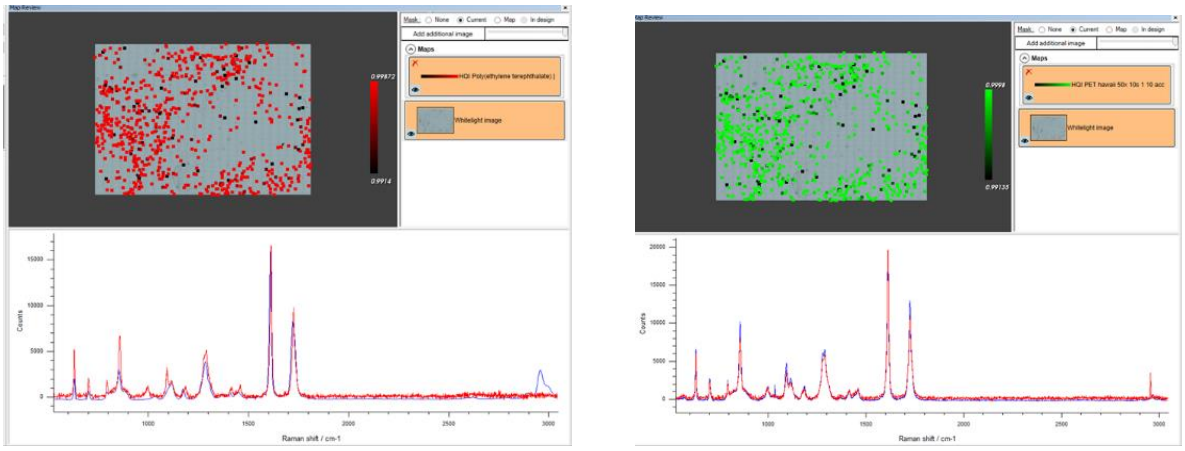

Figure S.2. Mapping of 774 PET particles with chemical identity recognition. The red squares show the spectrum compared to the reference PET (left) and the Hawaii PET (right). Black squares are non-focus PET particles to which the database gives a lower score in identification.

Table S.1: Number of PET and non-PET particles of the size limit 30 µm and more (size expressed as Feret min diameter) in the units allocated to homogeneity.

Unit	Number of PET particles F_{min} ≥30 µm	Number of PET particles with Feret min diameter ≥31 µm	Number and chemical identity of non-PET particles with Feret min diameter >30 µm (n.i. = not identified; n.o. = natural origin: cellulose, skin, $CaCO_3$, TiO_2, dust etc.)
210	371	370	16 (3 PE + 4 PS + 1 polyorganosiloxan + 8 n.i.) + 11 (n.o.)
570	347	345	1 (n.i.) + 24 (n.o.)
435	425	424	3 (1 PTFE + 2 n.i.) + 27 (n.o.)
83	362	361	3 (2 PS + 1 PP) + 26 (n.o.)
506	386	384	4 (1 PP + 3 n.i.) + 25 (n.o.)
627	result rejected due to particle loss		
361	426	426	11 (1 PE + 1 PP + 1 POM + 8 n.i.) + 40 (n.o.)
161	494	494	6 (1 PE + 1 PS + 1 PVP + 3 n.i.) +72 (n.o.)
2	471	470	12 (1 PE +1 PP + 4 PTFE + 1 POM + 1 PVP + 4 n.i.) +53 (n.o.)
287	419	419	8 (1 PP + 1 PTFE + 6 n.i.) + 58 (n.o.)

Table S.2: Chemical identity of particles detected in blank samples (particle size threshold same as for the reconstituted water samples) during homogeneity assessment (n.i.=not identified).

Blank	PET	PE	PS	PP	Cellulose	Skin (polyamide)	Cellulose acetate	$CaCO_3$	n.i.	Sum
1	0	0	0	0	5	0	1	0	0	6
2	0	0	0	0	6	0	0	1	1	8
3	0	1	0	0	3	1	2	0	0	7
4	0	0	0	0	7	5	0	0	1	13
5	0	0	0	0	2	1	0	0	1	4
6	0	0	0	0	3	2	0	0	0	5
7	0	0	0	0	3	1	0	0	5	4
8	0	0	0	0	7	3	0	0	5	15
9	0	0	0	0	13	2	0	0	1	16
10	0	0	1	1	3	2	0	0	3	10

Table S.3: PET particle number and non-PET particle number in the units assigned to transport stability assessment.

Unit	Number of PET particles with Feret min diameter >30 µm	Number and chemical identity of non-PET particles with Feret min diameter >30 µm (n.i. = not identified; n.o. = natural origin: cellulose, skin, $CaCO_3$, TiO_2, dust etc.)
40	438	11 (1 PE + 1 PTFE + 9 n. i.) + 41 n.o.
183	379	89 (5 PE + 3 PP + 1 PS + 1 PTFE + 1 PVC + 76 n.i.) + 438 n.o.
314	414	32 (1 PE + 1 PVC + 5 PTFE + 25 n.i.) + 197 n.o.
482	381	14 (1 PE + 1 PMMA + 2 polysiloxane + 3 PS + 7 n. i.) + 107 n.o.
619	411	50 (1 PE + 38 polysiloxane + 1PS + 10 n.i.) + 105 n.o.
130	480	21 (1 PE + 1 PTFE + 19 n.i.) + 113 n.o.
246	463	17 (2 PE + 1 POM + 1 PS + 1 PVC + 12 n. i.) + 64 n.o.
412	409	1 (1 PE) + 2 n.o.
539	366	10 (1 PE + 1 PS + 1 polysiloxane + 7 n.i.) + 66 n.o.
693	309	23 (1 PP + 5 PTFE + 1 polysiloxane + 16 n.i.) + 121 n.o.

Table S.4: Chemical identity of particles detected in blank samples (particle size threshold same as for the reconstituted water samples) during transport stability assessment.

Blank #	PET	PE	PS	PP	PTFE	Silicone	Cellulose	Skin (polyamide)	Cellulos eacetat e	n.i.	Sum
1	0	0	0	0	0	0	9	6	0	2	17
2	0	0	2	0	0	0	12	12	0	9	35
3	0	0	0	0	1	0	9	12	0	15	37
4	0	0	1	0	0	0	11	8	0	16	36
5	0	0	0	0	3	1	0	6	3	10	23
6	0	0	0	0	0	0	6	0	2	4	12
7	0	0	1	1	1	0	26	18	0	14	61
8	0	0	0	0	0	0	8	18	0	16	42
9	0	0	0	0	0	0	17	8	1	22	48
10	0	0	0	0	0	0	5	33	4	18	60

Table S.5: PET and non-PET particle numbers in the candidate RM units allocated to the storage stability study.

Unit	Number of PET particles with Feret min diameter >30 µm	Number and chemical identity of non-PET particles with Feret min diameter >30 µm (n.i. = not identified; n.o. = natural origin: cellulose, skin, $CaCO_3$, TiO_2, dust etc.)
112	474	10 (2 PE + 2 polysiloxane + 3 PS + 3 n.i.) + 57 n.o.
421	317	31 (1 PCL + 8 PVP + 1 polysiloxane + 2 PS + 19 PTFE) +71 n.o.
546	439	26 (1 PP + 1 PE + 2 PS + 22 n.i.) + 129 n.o.
180	500	18 (1 PMMA + 1 polysiloxane + 1 POM + 1 PS + 14 n.i.) + 125 n.o.
297	474	5 (1 polysiloxane + 4 n.i.) + 72 n.o.
32	441	15 (2 PS + 13 n.i.) + 81 n.o.
315	489	16 (1 PS + 1 PTFE + 14 n.i.) + 131 n.o.
485	403	27 (1 polysiloxane + 1 PS + 25 n.i.) + 145 n.o.

Table S.6: Chemical identity of particles detected in blank samples (particle size threshold same as for the reconstituted water samples) during storage stability study.

Blank #	PET	PE	PS	PP	PTFE	Silicone	Cellulose	Skin (polyamide)	Cellulose acetate	n.i.	Sum
1	0	0	0	0	0	0	0	0	0	0	0
2	0	0	0	0	0	0	35	14	1	7	57
3	0	0	0	0	0	0	14	24	3	12	53
4	0	0	2	0	0	0	16	8	2	6	34
5	0	0	0	0	0	4	8	22	0	9	43
6	0	1	3	0	0	0	11	18	0	14	47
7	0	0	0	0	0	0	12	9	5	17	43
8	0	0	1	0	0	0	6	5	4	6	22

Table S.7: PET and non-PET particle numbers in the candidate RM units allocated to evaluation of small particles' content.

Unit #	Number of PET particles with Feret min diameter 10-50 µm	Number of PET particles with Feret min diameter 1-10 µm	Number of particles with Feret min diameter 10-50 µm other than PET	Chemical identity of non-PET particles with Feret min diameter 10-50 µm (n.i. = not identified)
229	24	1218	408	2 PE, 2 PP, 3 PS, 1 PTFE, 6 silicone, 394 n.i.
104	10	889	736	1 CA, 1 PMMA, 1 PP, 2 PS, 2 PTFE, 1 PVC, 8 silicone, 720 n.i.
679	28	1269	1560	1 PE, 1 PTFE, 4 PVC, 17 silicone, 1537 n.i.
318	26	724	580	1 PE, 1 PLA, 4 PP, 2 PS, 6 PTFE, 19 silicone, 547 n.i.
576	33	500	608	1 PE, 1 PP, 2 PS, 8 PTFE, 1 PVC, 30 silicone, 565 n.i.
364	30	559	606	2 CA, 1 PE, 10x PP, 4 PS, 3 PTFE, 1 PVC, 587 n.i.

Table S.8: The number and the chemical identity of non-PET particles in blank samples (particle size threshold same as for the reconstituted water samples) during evaluation of small particles' content.

Number & (size range)	PET	PE	PS	PP	PTFE	PLA	silicone	cellulose	Skin (poly amide)	Cellulose acetate	n.i.
1 (10-50 µm)	0	0	0	3	1	0	17	26	30	0	106
1 (1-10 µm)	0	145	0	145	0	0	435	435	725	145	138830
2 (10-50 µm)	0	0	0	3	0	0	18	12	21	0	312
2 (1-10 µm)	0	0	0	0	0	816	408	408	4084	5309	154786
3 (10-50 µm)	0	0	0	1	0	0	2	11	17	0	256
3 (1-10 µm)	0	0	0	0	0	708	0	708	1772	354	218685
4 (10-50 µm)	0	0	0	0	0	0	11	43	17	0	94
4 (1-10 µm)	0	0	0	0	0	0	397	397	994	0	66255
5 (10-50 µm)	0	0	1	7	0	0	2	36	17	0	145
5 (1-10 µm)	216	216	216	0	0	216	0	216	866	2165	82715
6 (10-50 µm)	0	1	0	2	8	0	2	23	53	0	213
6 (1-10 µm)	0	498	0	0	0	3491	0	0	5986	12471	175591

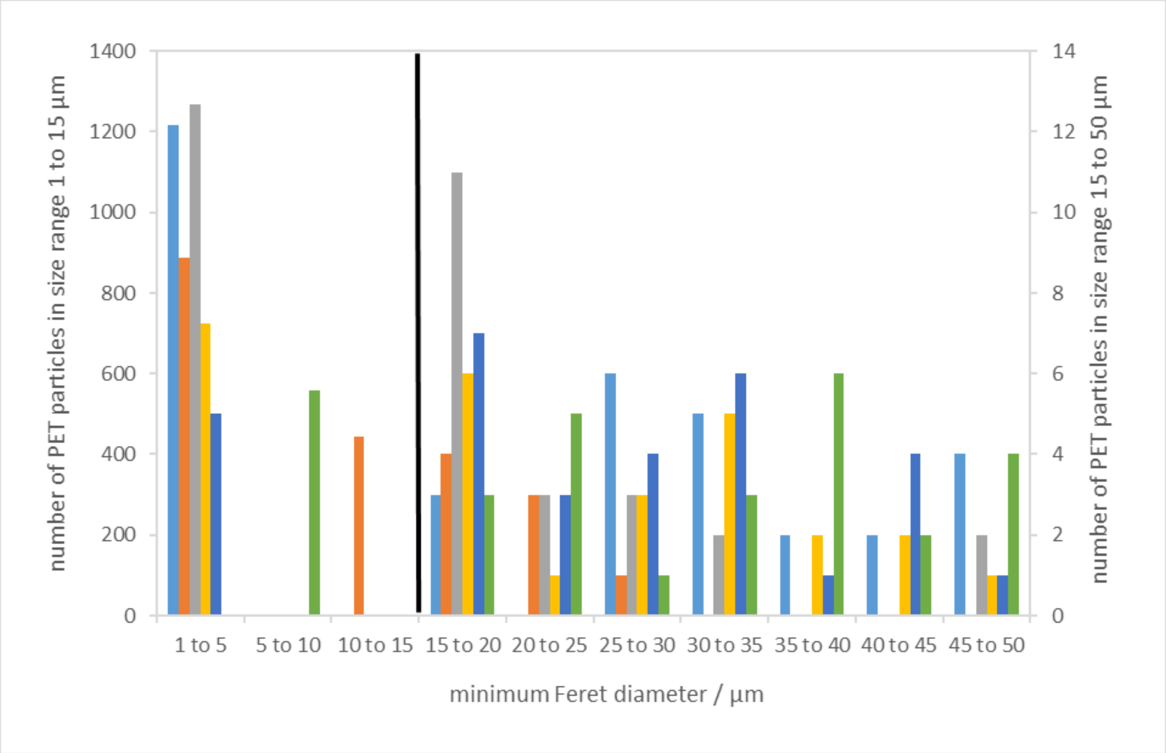

Figure S.3. PET particle size distribution in the units assigned to Evaluation of small particles' content.

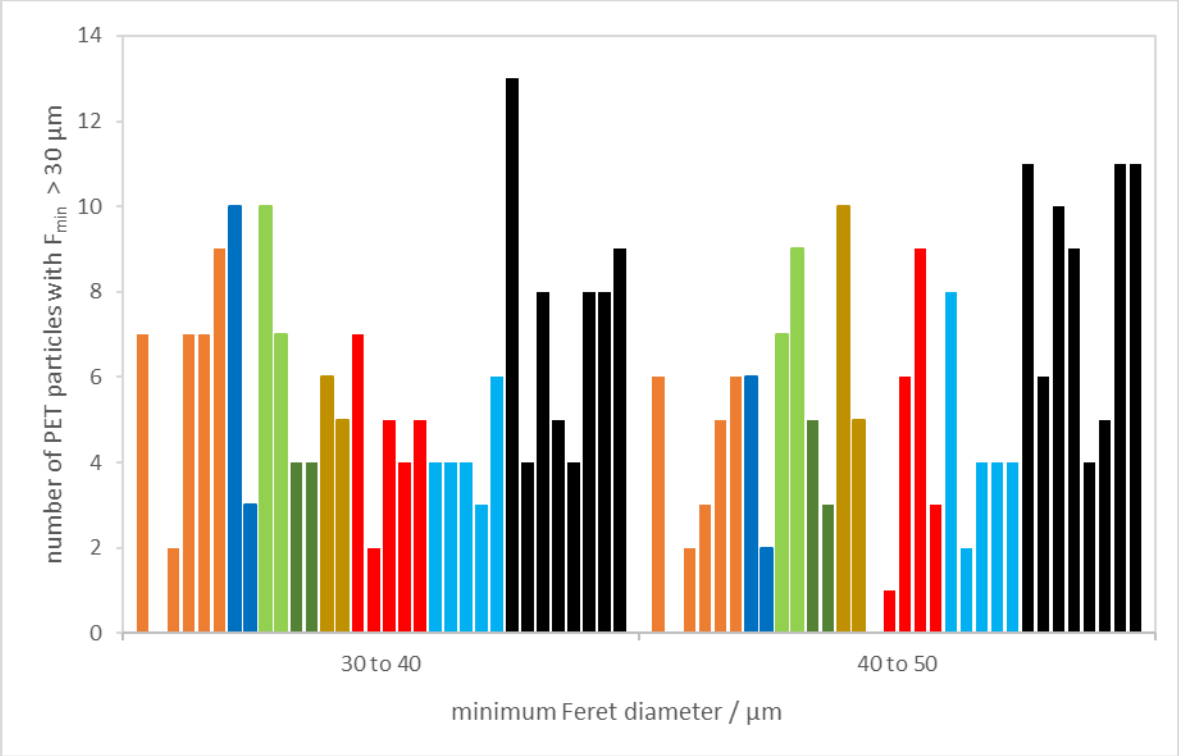

Figure S.4: PET particle number concentration in all units of the candidate RM, covering only particles in the size range 30-40 µm and 40-50 µm. Each colour represents one series of measurements (homogeneity, stability, evaluation of small particles' content).

Table S.9: Non-PET polymer particles detected in the candidate RM units allocated to homogeneity assessment.

Particle #/unit	F_{min} / µm	F_{max} / µm	Aspect ratio	Area / µm²	Thick-ness / µm	Optical image
77/2 PTFE	60	66	1.42	3004	15	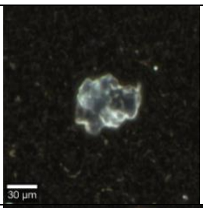
68/210 PS	80	316	3.95	2546	38	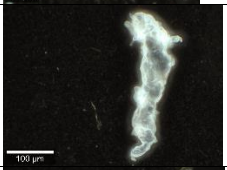
131/210 PE (fibre)	14	360	25.7	744	40	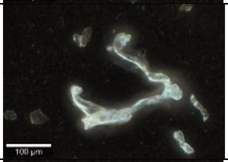
186/506 PP	38	63	1.67	6691	9	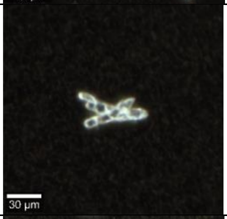
312/361 POM	108	311	2.88	15176	44	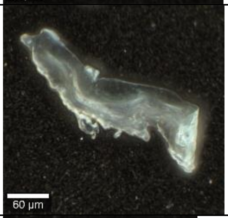
133/161 PVP	53	59	1.11	2394	32	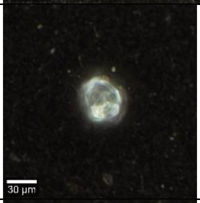
651/210 Polyorgano-siloxane	31	36	1.16	930	20	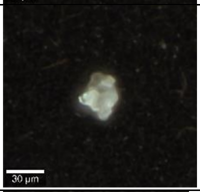
9/210 not identified (dust)	35	68	1.9	1653	8	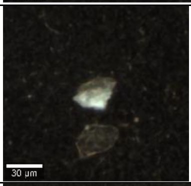
267/435	45	52	1.17	1011	12	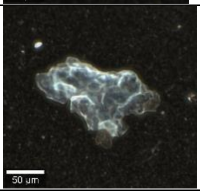
